# Associations between ozone and morbidity using the Spatial Synoptic Classification system

**DOI:** 10.1186/1476-069X-10-49

**Published:** 2011-05-24

**Authors:** Adel F Hanna, Karin B Yeatts, Aijun Xiu, Zhengyuan Zhu, Richard L Smith, Neil N Davis, Kevin D Talgo, Gurmeet Arora, Peter J Robinson, Qingyu Meng, Joseph P Pinto

**Affiliations:** 1 Institute for the Environment, The University of North Carolina at Chapel Hill, Chapel Hill, North Carolina, 27599, USA; 2 Gillings School of Global Public Health, The University of North Carolina at Chapel Hill, Chapel Hill, North Carolina, 27599, USA; 3 Department of Statistics, Iowa State University, Ames, Iowa, 50011, USA; 4 Department of Statistics and Operations Research, The University of North Carolina at Chapel Hill, Chapel Hill, North Carolina, 27599, USA; 5 HSBC Finance Corporation, Elmhurst, Illinois, 60126, USA; 6 Department of Geography, The University of North Carolina at Chapel Hill, Chapel Hill, North Carolina, 27599, USA; 7 School of Public Health, University of Medicine and Dentistry of New Jersey, Piscataway, New Jersey, 08854, USA; 8 U.S. Environmental Protection Agency, Research Triangle Park, North Carolina, 27711, USA

## Abstract

**Background:**

Synoptic circulation patterns (large-scale tropospheric motion systems) affect air pollution and, potentially, air-pollution-morbidity associations. We evaluated the effect of synoptic circulation patterns (air masses) on the association between ozone and hospital admissions for asthma and myocardial infarction (MI) among adults in North Carolina.

**Methods:**

Daily surface meteorology data (including precipitation, wind speed, and dew point) for five selected cities in North Carolina were obtained from the U.S. EPA Air Quality System (AQS), which were in turn based on data from the National Climatic Data Center of the National Oceanic and Atmospheric Administration. We used the Spatial Synoptic Classification system to classify each day of the 9-year period from 1996 through 2004 into one of seven different air mass types: dry polar, dry moderate, dry tropical, moist polar, moist moderate, moist tropical, or transitional. Daily 24-hour maximum 1-hour ambient concentrations of ozone were obtained from the AQS. Asthma and MI hospital admissions data for the 9-year period were obtained from the North Carolina Department of Health and Human Services. Generalized linear models were used to assess the association of the hospitalizations with ozone concentrations and specific air mass types, using pollutant lags of 0 to 5 days. We examined the effect across cities on days with the same air mass type. In all models we adjusted for dew point and day-of-the-week effects related to hospital admissions.

**Results:**

Ozone was associated with asthma under dry tropical (1- to 5-day lags), transitional (3- and 4-day lags), and extreme moist tropical (0-day lag) air masses. Ozone was associated with MI only under the extreme moist tropical (5-day lag) air masses.

**Conclusions:**

Elevated ozone levels are associated with dry tropical, dry moderate, and moist tropical air masses, with the highest ozone levels being associated with the dry tropical air mass. Certain synoptic circulation patterns/air masses in conjunction with ambient ozone levels were associated with increased asthma and MI hospitalizations.

## Background

The complex relationship between climate, air pollution, and health outcomes has been explored in many research studies that characterize health outcomes in terms of correlates of meteorological and air quality variables. It is well known that temperature is a good predictor of morbidity and mortality, especially during extremes of cold or heat, or seasonally [1-5]. In addition, exacerbation of asthma symptoms has been linked to day-to-day variations in temperature and humidity [6] and to thunderstorms [7].

Extensive research indicates that exposure to air pollution has serious public health consequences in terms of both morbidity and mortality. Epidemiologic studies indicate that elevated levels of ground-level ozone are linked to cardiovascular and respiratory morbidity and mortality [8-12]. In particular, the published literature [8] shows that subpopulations with cardiovascular disease or respiratory disease are more likely to be affected by air pollution. This ozone-mortality association, however, shows regional heterogeneity (based on intercity response), with cities in the northeastern United States showing greater response [13]. Several studies [8-14] have shown that the risk of hospitalization increases with elevated levels of ozone (and also with elevated levels of the fine particulate matter known as PM_2.5_). However, our understanding of the relationships among meteorological parameters, air pollution, and health remains incompletely defined [15-18].

Ozone in the atmosphere is a secondary pollutant: it is formed by photochemical reactions of precursors, rather than being directly emitted from specific sources. Ozone and other oxidants, such as peroxyacyl nitrates and hydrogen peroxide (H_2_O_2_), form in polluted areas by atmospheric reactions involving two main classes of precursor pollutants: volatile organic compounds (VOCs) and nitrogen oxides (NO_x_).

The chemical processes and meteorological conditions involved in ozone formation typically extend over spatial scales of hundreds of thousands of square kilometres [19,20], which suggests that ozone formation is best understood in the context of large-scale atmospheric patterns and not just in terms of local conditions.

Usually when associations between adverse health effects and air pollutants are studied, only a few meteorological variable are considered (for example, temperature and relative humidity). However, additional information can be gained when examining these associations by including additional meteorological variables relevant to air pollution development, as described above. One such approach involves the use of air masses. An air mass is defined as a large volume of air with relatively homogenous meteorological variables (temperature, humidity, cloud cover, etc.) that occurs over large spatial domains, usually for several days. The relationships among meteorological parameters characterize the identity of air masses in terms of dry versus wet and warm versus cold, in association with other related parameters such as cloudiness, precipitation, and winds. Thus, characterization of weather at a given location using the air mass concept also provides information about the potential of meteorological conditions to affect air quality, the transport history of the air, and the upwind pollution sources affecting air quality on a particular day at that given location.

The Spatial Synoptic Classification (SSC) system [21,22] has been used to characterize meteorological conditions at various locations in terms of air masses. Pope and Kalkstein [23] proposed a synoptic climatology approach to account for the possible confounding effect of weather on the relationship between course particulate matter (PM_10_) and mortality. Subsequently, the synoptic approach has been used in a number of studies examining linkages between weather situations and health outcomes [21,24-26], and between weather situations, air pollution [27], and health outcomes [28,29]. These studies were able to place results in a broader context than would have been obtained by using individual meteorological variables.

In this research we use the air mass approach to examine the complex relationship between meteorology and air quality and the associated health outcomes. The question that we address here is how changes (increases or decreases) in asthma and/or myocardial infarction (MI) hospital admissions are related to changes in concentrations of ozone for a specific air mass. We use the SSC approach to characterize meteorological conditions in five cities in North Carolina, in terms of seven air mass types over a 9-year period (1996 through 2004). We then examine the associations between day-to-day variations in air masses, levels of ozone, and hospital admissions for asthma and MI over the same 9-year time frame.

## Methods

### Exposure

We used daily meteorological, air quality, and hospitalization data for 1996 through 2004 for five cities in North Carolina: Asheville, Charlotte, Greensboro, Raleigh, and Wilmington. Charlotte is the largest metropolitan area in the state, followed by Raleigh (the capital city) and Greensboro. These three cities are located in the central part of North Carolina. Asheville is located to the west, at relatively high altitude (elevation 650 m), and Wilmington is located on the coast. Data for daily maximum temperature, daily average dew point temperature, daily average sea-level pressure, and daily maximum 1-hour ozone were obtained from the U.S. EPA Air Quality System (AQS) [30]. Daily hospital admissions for asthma and myocardial infarctions for the 1996-2004 analysis period were obtained from the North Carolina Department of Health and Human Services (DHHS) State Center for Health Statistics. Meteorological, air quality, and hospitalization data were then linked by date.

The Spatial Synoptic Classification (SSC) system [21,22] takes in surface weather data from a given meteorological station and classifies each day into one of the following seven weather types or air masses, based on dry or moist conditions and on air mass origin as either polar, midlatitude, or tropical: dry moderate (DM), dry polar (DP), dry tropical (DT), moist moderate (MM), moist polar (MP), moist tropical (MT), and transitional (TR). The MT type is further broken down into its extremes, the moist tropical + (MT+) and the moist tropical ++ (MT++) air masses, which are found on the warmest and most humid MT days. The classification of air mass applies to conditions at a specific location and could differ from one location to another on the same day, subject to the prevailing synoptic weather conditions and terrain. The proximity of cities with similar conditions may result in similarity of the daily air mass pattern.

Synoptic air mass classifications for each day, for each of the five cities, for the period from January 1, 1996, through December 31, 2004, were based on the analysis method of Sheridan [31]. In the SSC method, "seed days" representing days with the typical meteorological character of each air mass at a particular location are chosen for different times of the year. Discriminant function analysis is used to produce equations for each air mass (based on the "seed days" that were chosen) that are used to assign each of the other days to a particular air mass type. Applying the SSC system results in the classification of each day at a meteorological station into a specific air mass, based on temperature, dew point, pressure, wind speed and direction, cloud cover, and cloud opacity. We created box plots for daily maximum 1-hour ozone, daily maximum temperature, daily average dew point temperature, and daily average sea-level pressure for each air mass for each of the five cities.

To provide further analysis of the air quality characteristics of various air masses, we analyzed the relationship for the city of Charlotte between air mass ("AM") and ozone ("O3") occurrences with four distinct probabilities (these probabilities are associated with each other under Bayes' Law).

• P(O3|AM)--The conditional probability of the ozone concentration being above a certain level given that a day is governed by a certain air mass.

• P(AM|O3)--The conditional probability of the appearance of a certain air mass given a day with ozone concentration above a certain level.

• P(O3)--The marginal probability of finding ozone concentration above a given level.

• P(AM)--The marginal probability of the appearance of a certain air mass.

To identify potential pollution source regions that are upwind of Charlotte for different air masses, we calculated 72-hour backward trajectories using the Hybrid Single Particle Lagrangian Integrated Trajectory (HYSPLIT) model [32]. We analyzed the frequency of 72-hour backward trajectories of near-surface flow for each air mass during the spring and summer of 2001 through 2003 for Charlotte. The number of times trajectories crossed each 1- × 1-km grid cell in a domain consisting of the contiguous U.S., Canada, and Mexico was divided by the total number of trajectories crossing all the grid cells in the domain to give a percentage. The proximity of Charlotte, Raleigh, and Greensboro led us to conclude that the trajectory analysis conducted for Charlotte is representative for the other two cities as well. We focused the analysis on the three air mass types that were characterized by the highest concentrations of ozone--the dry moderate (DM), dry tropical (DT), and moist tropical (MT) air masses--to identify the likely sources associated with each air mass.

### Measures of Respiratory and Cardiovascular Morbidity

Daily hospital admissions data were obtained from the North Carolina Department of Health and Human Services (DHHS), with assistance from North Carolina's State Center for Health Statistics (SCHS), and covered the years 1996-2005. The SCHS is legislatively mandated to maintain copies of hospital discharge data from all hospitals in North Carolina. The discharge data include information on all in-patient hospital stays from all of the state's non-Federal, short-stay general and specialty hospitals. The data do not include information from the state's seven psychiatric hospitals. Hospitalizations with ICD-9 codes for myocardial infarction (410.x) and asthma (493.x) were selected to represent both cardiovascular and respiratory disease morbidity. Hospitalization data include individuals of all ages (0-100) and all races.

Using a generalized linear model (GLM), we modeled the association between daily maximum 1-hour ozone and hospitalizations for asthma and MI for the various air mass types, adjusting for meteorology, seasonality, and long-term trend. More specifically, if the number of deaths on day *t *follows a Poisson distribution with mean μ_*t*_, we assume a relationship of form

where the β_*j *_are unknown coefficients, and x_*j,t *_are covariates. Specifically, the covariates include ozone, an indicator variable representing air-mass type, average daily dew-point temperature, an indicator variable representing day of week, and terms representing the seasonal and long-term trend effects. The latter are modeled by B-splines, which is a common mathematical technique for representing a smooth nonlinear function as a linear function of fixed basis functions [33]. The number of basis functions used (or knots) is to some extent arbitrary, but air pollution studies often use between 3 and 10 knots per year, to represent a balance between including enough knots to represent seasonal effects but not so many that they become confounded with the air pollution effects that we are trying to model. In the present study, we used a total of 24 knots.

Health effects may occur not just immediately after an increase in pollution level, but may be spread out over several days [34-36]. We studied the lag effects by fitting models using lags of up to 5 days. To assess the validity of the Poisson assumption, we estimated the overdispersion parameter using quasi-likelihood method and found no significant violation of the Poisson assumption. There were no overdispersion problems: all of the overdispersion parameters were around 1.

## Results

**Figure 1:**
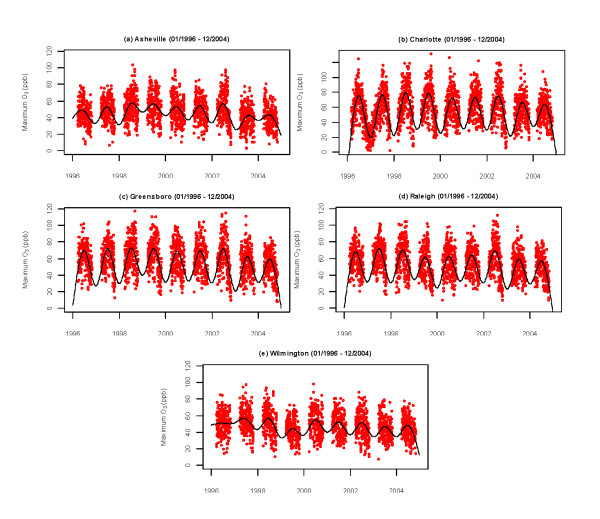
**Daily maximum 1-hour ozone concentrations (during the ozone season, April-September) for (a) Asheville, (b) Charlotte, (c) Greensboro, (d) Raleigh, and (e) Wilmington**. The solid line represents a simple natural spline smoother to highlight the overall trend.

Daily maximum 1-hour ozone time series (1996-2004) for the five cities are shown in Figure 1; the data are plotted for the ozone season (April through September). The figure shows pronounced summer maxima measured in all of the cities, although the magnitude of the seasonal variation varies from city to city. In addition, maximum O_3 _concentrations are typically above 100 ppb, except in Asheville. Table 1 shows the annual 95^th ^percentile for the data for each city. In general, the larger urban areas of Charlotte, Raleigh, and Greensboro have more days with daily maximum 1-hour ozone exceeding 100 ppb than do Wilmington and Asheville. The years 2003 and 2004 show lower daily maximum 1-hour ozone values for the five cities compared to other years. These lower values could be related to large-scale patterns, as 2003 was an El Niño year. However, 1997 was also an El Niño year, but does not show lower values, even though the El Niño that year was stronger. Other factors such as changes in emissions might also be involved.

**Table 1:** Annual 95^th ^percentiles of daily maximum 1-hour ozone (ppb)

City	1996	1997	1998	1999	2000	2001	2002	2003	2004
**Charlotte**	89	91	102	103	92	94	101	83	80

**Raleigh**	83	91	90	83	81	80	93	78	72

**Greensboro**	84	87	96	94	89	89	96	79	74

**Asheville**	67	69	79	78	76	70	81	60	63

**Wilmington**	72	76	76	64	74	71	72	70	67

**Figure 2:**
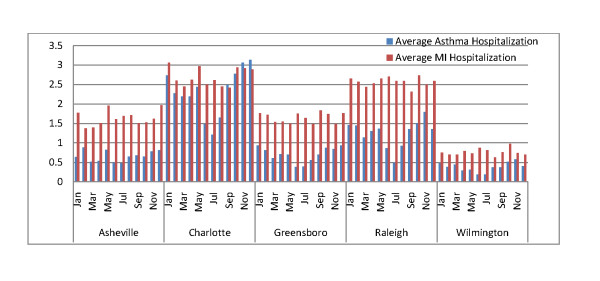
**Monthly average hospital admissions rate (per day) for asthma and myocardial infarction for the five cities**.

The seasonal distribution of daily hospital admission rates for the nine years of data considered for the study is shown in Figure 2. The asthma and MI daily hospital admission rates are the highest for Charlotte, followed by Raleigh, due to the relatively large populations of these cities. The hospitalization rate for MI is almost always higher than for asthma in all five cities. The asthma hospitalization rates depict a seasonal pattern, with highest admissions during the fall and winter, while the MI hospitalization rates are more nearly constant throughout the year. Also noticeable are the relatively high asthma and MI hospital admission rates for Asheville compared to Wilmington (the two smaller cities).

### Air Mass Classifications in North Carolina

Besides the delineation of air mass properties in terms of their moisture and temperature characteristics, each one relates to a particular synoptic-scale pattern, as revealed by the daily weather map [37]. With a focus on North Carolina, we briefly identify the surface and middle troposphere meteorological patterns that characterize each air mass. The dry polar (DP) portrays a middle-troposphere deep low-pressure trough associated with a surface high pressure that brings very cold temperatures and dry conditions to the state. The jet stream migrates to lower latitudes. A middle-troposphere cutoff low, to the west of North Carolina, displaces this pattern for the moist polar (MP), which brings to the state cloudy, humid, and cool conditions associated with northeasterly flow from the Atlantic. The dry tropical (DT) and dry moderate (DM) air masses for North Carolina portray similar synoptic circulation patterns but with different moisture and temperature conditions, with surface high pressure over the southeastern U.S. associated with the subsidence of air that undergoes warming and drying after passing over the Appalachian Mountains. In addition to temperature and moisture differences, the DT and DM air masses can be distinguished from each other, in North Carolina, based on their near-surface flow patterns: the DT air mass is mainly associated with southwesterly flow that does not originate from the Gulf of Mexico, while the DM air mass is associated with more northeasterly or northwesterly flow. The moist moderate (MM) air mass is mainly associated with more easterly or northeasterly flow bringing relatively cool and moist air and precipitation to North Carolina. The moist tropical (MT) air mass brings to North Carolina warm and humid conditions associated with near-surface southerly flow from the Gulf of Mexico and zonal (E-W) flow aloft. Its extremes, MT+ and MT++, are distinguished by a surface high-pressure system over the Atlantic to the east. The transitional (TR) air mass describes the situation on days when one air mass gives way to another.

**Figure 3:**
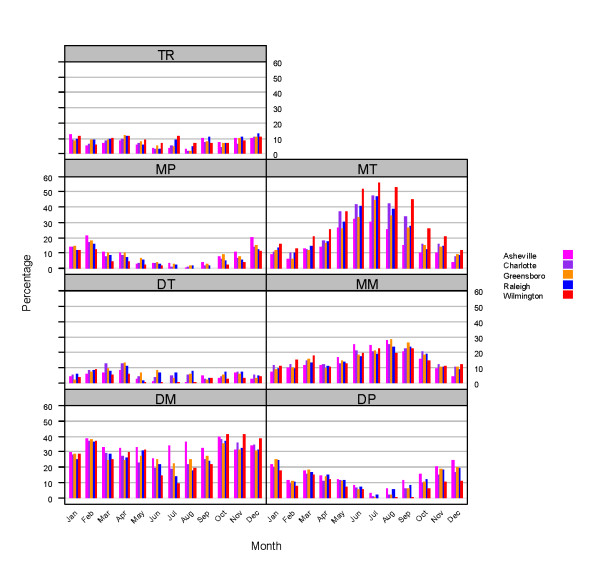
**Monthly frequency of occurrence of seven air mass types based on daily meteorological analyses for 1996-2004**.

Figure 3 shows the monthly frequency of occurrence of the seven air mass types for each of the five cities in North Carolina, for the analysis period (1996-2004). The frequency of occurrence of some of these air masses shows large seasonal variations. The DP and MP air masses prevail during the fall and winter months. The MT air mass also shows a clear seasonal pattern with a summer peak for all cities, with Wilmington showing distinctly higher values of occurrence than the other cities. The DM maximum occurrence is during the fall and winter, and it also has the second highest occurrence, behind the MT and MM air masses, during April to September. For Asheville (mountain city), however, the DM air mass shows steady occurrence throughout the whole year. Noticeable also is the low but steady occurrence of the DT air mass throughout the entire year for the five cities. The largest city-to-city differences occur during the summer and early fall for the DM and MT air masses. Maximum differences are between Asheville and Wilmington, because their climates are related to their geographic locations; Asheville is characterized by a temperate mountain climate, while Wilmington has a semitropical coastal climate.

### Air Quality and Meteorological Characteristics of Air Masses

**Figure 4:**
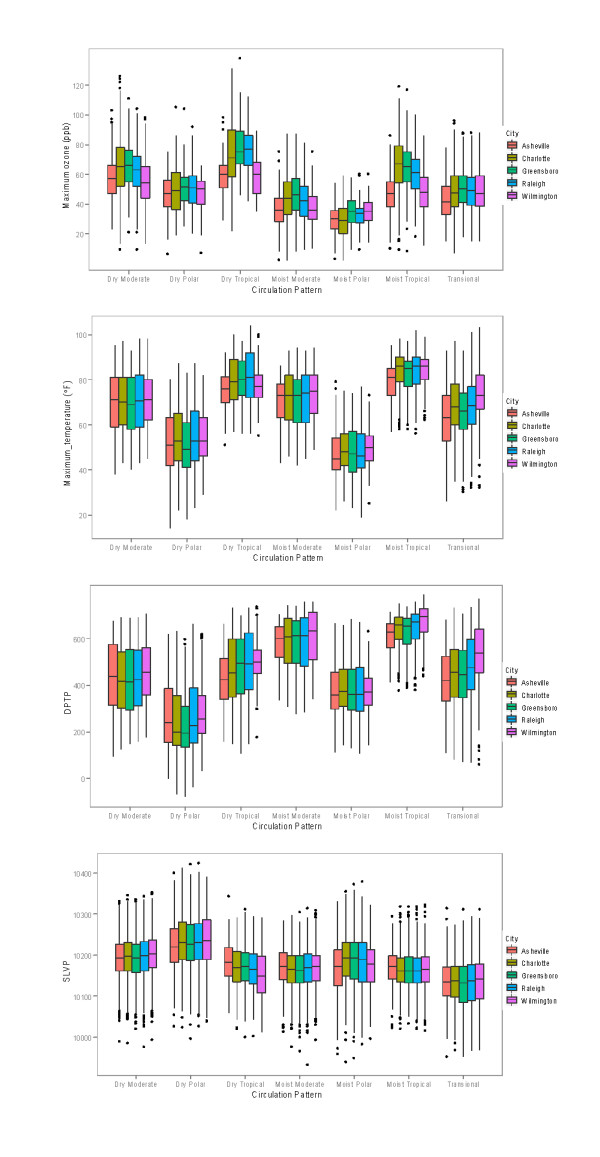
**Meteorological and air quality characteristics of the seven air mass types**. Box plots represent median, interquartile range, and overall range of daily maximum 1-hour ozone (ppb), daily maximum temperature (°F), daily average dew point temperature (DPTP, °F), and daily average sea-level pressure (SLVP, tenths of millibar).

Selected meteorological and air quality characteristics of each air mass for each of the five cities are given in Figure 4, which shows box plots for daily maximum 1-hour ozone, daily maximum temperature, daily average dew point temperature, and daily average sea-level pressure. The figure reveals clear distinctions between air masses, with substantial differences in ozone levels across air masses. Similar results were shown by Davis et al. [27] for locations in the Washington, DC, vicinity and in West Virginia. The figure also reveals the clear similarity of meteorological and air quality properties for each air mass type when compared across the cities (i.e., when compared across each group of five bars). The median, maximum, and minimum values for each air mass variable are in general very similar for all of the cities, in particular for the three largest urban cities (Charlotte, Raleigh, and Greensboro), but also for Asheville (mountain city) and Wilmington (coastal city). The dry tropical and moist tropical air masses capture the highest daily maximum 1-hour ozone values and daily maximum temperature values for each city, followed closely by the dry moderate air mass. The DT air mass, as expected, is characterized by smaller values for the daily average dew point temperature than the MT air mass, whereas the MT air mass shows higher values of daily average dew point temperature. On the other hand, the DP and MP air masses occur at the lower end of the spectrum for daily maximum 1-hour ozone, daily maximum temperature, and daily average dew point temperature.

**Figure 5:**
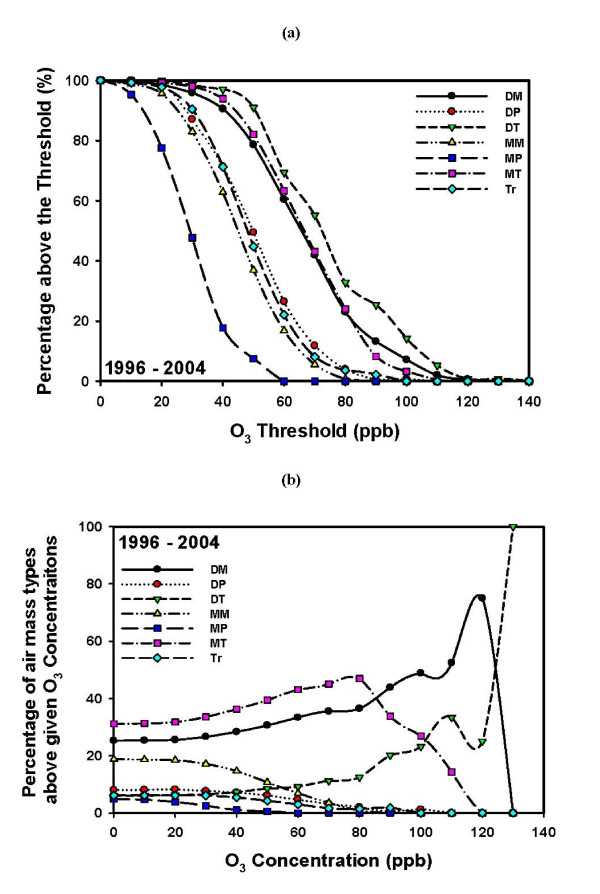
**Ozone distribution of the seven air masses for the 9-year period 1996-2004**. (a) Probability (expressed as a percentage) of finding ozone (O_3_) concentrations above a threshold concentration for a given air mass (P(O_3_|AM)). (b) Probability (expressed as a percentage) of having a particular air mass present when ozone concentrations are above a threshold concentration (P(AM|O_3_)).

Figure 5a shows the probability of finding ozone above a threshold concentration for each air mass [P(O3|AM), discussed in the "Methods" section]. This probability is highest for the dry tropical air mass, followed by the moist tropical and dry moderate air masses. As was shown in Figure 3, the DT air mass occurs less frequently than the MT and DM air masses. The highest ozone levels (>80 ppb) distinguished the DT, DM, and MT air masses. Lower ozone values are associated with the other air masses (dry polar, moist polar, moist moderate, and transitional between air masses).

Figure 5b shows the probability of being under the influence of a particular air mass for a given ozone threshold concentration [P(AM|O3)]. The figure reveals an interesting pattern: for the 9-year period from 1996 through 2004, about 50% of days with an ozone concentration of ≥80 ppb were found under the MT air mass, 30% were found under the DM air mass, and 10% under the DT air mass. Higher ozone values (more than 100 ppb and 120 ppb) were associated with the presence of the DT and DM air masses. We note that while the DT and MT air masses have similar extreme temperature and ozone concentration characteristics, they differ widely in moisture content, as was shown in Figure 4.

**Figure 6:**
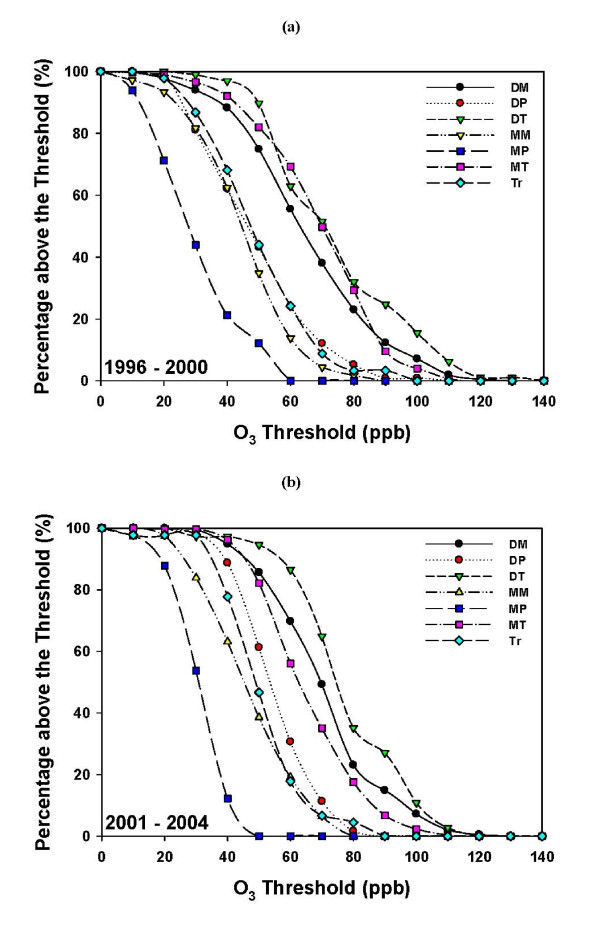
**Ozone distribution of the seven air masses**. (a) The 5-year period 1996-2000 and (b) the 4-year period 2001-2004. Probability (expressed as a percentage) of finding ozone (O_3_) concentrations above a threshold concentration for a given air mass (P(O_3_|AM)).

The above analyses utilize data from the entire 9-year period to produce the results shown. It is of interest to see whether such findings would also be true for subsets of the analysis period. Figure 6 shows the same statistics revealed in Figure 5 but broken down into one five-year and one four-year period (1996-2000 and 2001-2004). As can be seen, there were notable shifts in the relative importance of different air masses associated with high ozone. During the first (five-year) period (Figure 6a), ozone up to ~85 ppb was associated with either the DT or MT air masses, but above this level ozone was more likely to be found under DT or DM air masses. During the second (four-year) period (Figure 6b), the MT air mass decreases in importance compared to the DT and DM air masses above about 60 ppb. Although not shown, there were much larger changes from year to year.

High ozone levels are usually thought to be found mainly under the hot, humid conditions characteristic of the MT air mass. Instead, in Charlotte they are more likely to be found under the drier conditions associated with the DT and DM air masses. That is due to the complex relationship among ozone concentration, temperature, and emissions [38] and the possible advection of ozone precursors from upwind sources. As will be shown in the next section, air paths and trajectories associated with these air masses are more frequently from the southwest (for DT) and northwest/northeast (for DM) compared to the MT air mass, which is associated with more southerly flows bringing air from the Gulf of Mexico and Atlantic Ocean. The analyses confirm that the DT, DM, and MT air masses favor conditions of elevated ozone levels in North Carolina.

### Characteristics of Air Mass Trajectories

**Figure 7:**
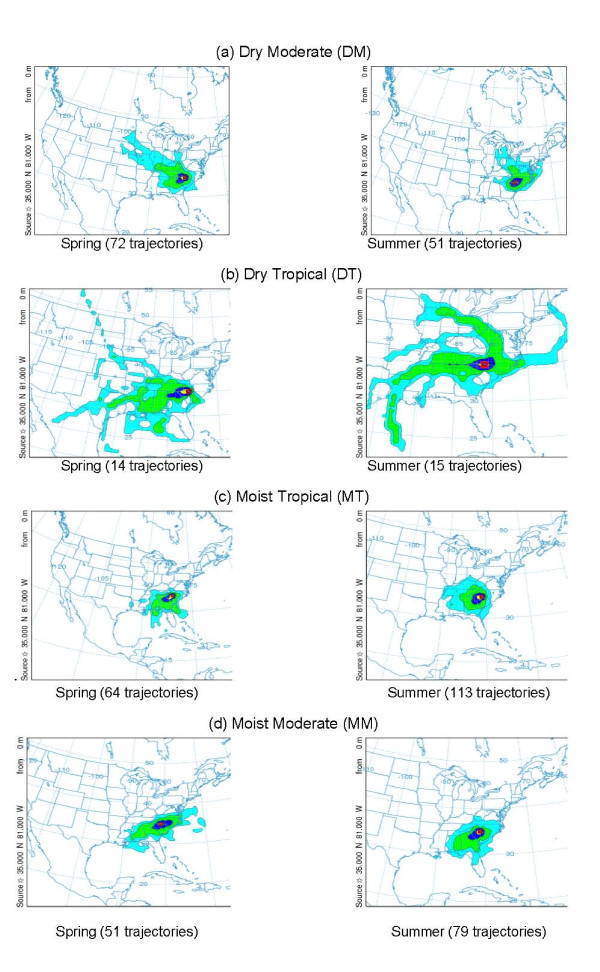
**Likely flow history of air masses during the spring and summer of 2001, 2002, and 2003 for Charlotte**. Frequency clusters of 72-hour backward trajectories, using EDAS (Environmental Data Analysis System) wind fields for different air masses: (a) DM air mass, (b) DT air mass, (c) MT air mass, and (d) MM air mass. The number of times trajectories crossed each 1- × 1-km grid cell over the contiguous U.S., Canada, and Mexico was divided by the total number of trajectories crossing all the grid cells to give a percentage.. Color legend: turquoise = 5%, green = 10%, blue = 25%, yellow = 50%.

An important aspect of the association between air quality and health impacts is the relevance of remote emissions sources that emit chemical species that can affect areas downwind. Davis et al. [27] have acknowledged the importance of linking trajectories to the type of air mass when studying high-ozone episodes. Figure 7 shows the paths taken by the 72-hour back trajectories from Charlotte for the DM, DT, and MT air masses. Figure 7 reveals that back trajectories for the DM air mass extend far to the northwest during spring, and to the northwest and northeast during summer. Back trajectories for the DT air mass extend even further back to the west in spring. In addition, in summer the DT air shows a trail of trajectories passing through the Ohio Valley, where many sources of NO_x _are located. MT air indicates, in general, a southerly flow from the Atlantic Ocean or the Gulf of Mexico during spring and summer. Also shown are the 72-hour backward trajectories for the MM air mass for comparison, even though it is not associated with elevated ozone due to its combined meteorological characteristics, as discussed above. Not shown are the back trajectories for the MP and DP air masses, due to their low probabilities of frequency during the spring and summer seasons. Note that because of the small number of back trajectories shown in Figure 7, the lower percentiles (10% and especially 5%) might represent at most only a few back trajectories, or even only one back trajectory.

### Association among Air Mass, Air Quality, and Hospitalizations

**Figure 8:**
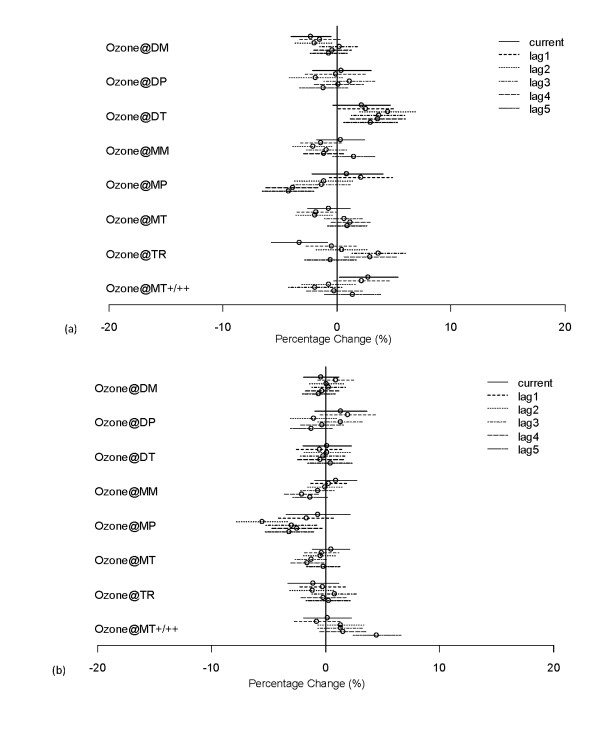
**The effect of short-term changes in ozone concentrations on hospital admissions under various air masses for Charlotte, Raleigh, and Greensboro for lags of 0 to 5 days**. Percentage change (and 95% confidence interval) in (a) asthma and (b) MI admissions per 10 ppb increase in ozone, controlling for dew point temperature, day of the week, nonlinear seasonal effects, and long-term trends.

Figures 8a and 8b show the percentage changes and 95% confidence intervals for asthma and myocardial infarction admissions per 10 ppb increase in ozone for various air masses, based on averaging the coefficients for Charlotte, Raleigh, and Greensboro; Asheville and Wilmington were excluded from the health outcome analyses because of a lack of statistical power and the much larger confidence limits found for Asheville and Wilmington compared to the three larger cities. Note also that the frequency of occurrence of different air masses is similar for Charlotte, Raleigh, and Greensboro but is different for Asheville and Wilmington (cf. Figure 3), because they are located in their own distinct climatic regions. Figure 8a reveals patterns of association between ozone increase and asthma hospital admissions for different air masses. For the DT air mass, increases in ozone result in lasting positive effects on hospital admission, with the percentage change being significantly larger than zero for all lags between 1 and 5. For the MT+/++ air mass, only the current-day effect is statistically significant, while for the TR air mass there is a delay of about three days in the increase in asthma admission, with the effects statistically significant at 3 days lag and 4 days lag. For the rest of the air masses, no statistically significant positive effects can be established at 5% significance level, though there is some weak evidence that the effects are positive under air masses MM, MP, and MT for some specific lags.

For MI hospital admissions (Figure 7b), the pattern of association with increased ozone level is quite different from that for asthma. The only statistically significant positive effect is for the MT+/++ air mass at 5 days lag, though there is some weak evidence that the effects are also positive starting from 2 days lag. Statistically significant associations were not found for the other air mass types.

## Discussion

We have presented results obtained by analyzing the association between ozone concentrations and hospital admissions for asthma and myocardial infarction in the general population in selected cities in North Carolina using meteorological, air pollution, and health data for nine years (1996-2004). We used the Spatial Synoptic Classification system to characterize daily meteorological conditions for the five cities during this 9-year period. Seven air mass types were based on dry or moist conditions and on air mass origin as either polar, midlatitude, or tropical. The analysis shows that each air mass has its own distinctive air quality and meteorological features. The analysis also shows the interaction between the meteorological variables (temperature, dew point, trajectories) and the air quality characteristics of each air mass. The dry moderate, dry tropical, and moist tropical air masses were characterized by the largest extremes of temperature and extremes of daily maximum 1-hour ozone concentrations exceeding specified threshold values. We further examined air mass characteristics for the city of Charlotte. Clusters of 72-hour back trajectories for Charlotte for spring and summer of 2001 through 2003 reveal different patterns corresponding to potential upwind source regions for the DM, DT, and MT air masses. The back trajectory analyses are particularly useful because they relate air masses to potential emission source regions upwind of a specific location. The back trajectory analyses indicate that the DM, DT, and MT air masses were associated with transport mainly from the northwest/northeast, southwest, and south, respectively. Thus, these air masses may be associated with different pollutant mixtures.

We extended the air mass/air pollution analysis to examine the association between ozone and morbidity due to asthma and MI daily hospitalizations for Charlotte, Raleigh, and Greensboro, using GLMs. The analysis showed statistically significant increases in asthma hospitalization with increases in ozone for the DT air mass for lags of 1 to 5 days, with near-significant increase for a lag of 0 days. The transitional (TR) and the extreme moist tropical (MT+/MT++) air masses also showed significant effects, but only at specific lags: lags 3 and 4, and same day, respectively. Statistically significant increases in asthma hospitalization as a result of increase in ozone levels were not found for the DM, MM, DP, and MP air masses. In general, statistically significant increases in hospital admissions for MI related to ozone increases were not found, with the exception of the MT+/MT++ air mass at 5 days lag. When considering asthma and MI hospital admissions together, only the extreme moist tropical air masses (MT+/++) showed any statistically significant association with both.

These results demonstrate that ozone-related increases in hospital admissions for asthma were associated with certain air mass types, while other types did not favor any increases in hospital admissions as result of increases in the ground-level ozone concentrations. Because the air masses affecting a particular location can be forecast as part of routine weather forecasting, the information resulting from the methodology described here could assist public health officials in planning effective resource allocations--for example, scheduling additional emergency room staff when the weather forecast indicates an upcoming specific air mass pattern that could affect the community's health. The concept of air mass classification could provide an important and distinctive approach to quantifying the complex association between climate/air quality and health, in particular for addressing possible health impacts under future climate scenarios. Note, however, that while multiple methodologies exist for classifying the synoptic-scale pattern at a specific location [29], it is imperative that any air-mass-based approach capture the synoptic-scale meteorological pattern to reduce the uncertainty in applying this methodology.

## Conclusions

Air mass approaches like the one presented here have the benefit of providing air quality and meteorological correlates in a systematic manner relevant to their potential impacts on health outcomes. For example, associations between pollutants and health outcomes could be different under different air masses, and adjusting single meteorological parameters cannot provide the potential association between air quality and health. On the other hand, the air mass approach may characterize situations more relevant to meteorological conditions (for example, heat waves) that contribute to aggravated health outcomes and not necessarily due only to, or associated with, elevated pollution levels. For example, Sheridan et al. [26] show the relationship between increase in mortality and the extreme levels of the DT and MT air masses at a number of cities in the United States. Different cities, at different locations, may show other specific types of air masses associated with elevated morbidity. A broader validation of the results of this research using data from cities across the U.S. with different weather systems is warranted.

We conclude that a synoptic air mass and trajectory analysis of the association between air pollution and morbidity can provide a better understanding of how atmospheric circulation patterns and air mass types affect pollution-associated respiratory and cardiovascular hospitalizations. The occurrence of individual air masses can be forecast, providing useful information for planning public health resources. This approach can also be applied to future climate scenarios, leading to a more comprehensive understanding and modeling of climatic change and air-pollution-related morbidity.

## List of abbreviations

DM: dry moderate; DP: dry polar; DT: dry tropical; GLM: generalized linear model; MI: myocardial infarction; MM: moist moderate; MP: moist polar; MT: moist tropical (its extremes are MT+ and MT++); P(AM): the marginal probability of the appearance of a certain air mass; P(AM|O3): the conditional probability of the appearance of a certain air mass given a day with ozone concentration above a certain level; P(O3): the marginal probability of finding ozone concentration above a given level; P(O3|AM): the conditional probability of the ozone concentration being above a certain level given that a day is governed by a certain air mass; SSC: Spatial Synoptic Classification; TR: transitional.

## Competing interests

The authors declare that they have no competing interests.

## Authors' contributions

AFH led the design of the study and analysis of the results, and drafted the manuscript. KBY participated in the design of the study, carried out the analysis of epidemiological data, and helped to draft the manuscript. AX carried out the meteorological analysis. ZZ designed the statistical model. RLS participated in the design of the statistical model. NND carried out the trajectory analysis of air masses. KDT participated in the trajectory analysis of air masses. GA performed the statistical analyses. PJR participated in the design of the study. QM participated in the analysis of air quality data. JPP participated in the design of the study and analysis of the results, and helped to draft the manuscript. All authors read and approved the final manuscript.

## References

[B1] O'Neil MS Zanobetti A Schwartz J Modifiers of the temperature and mortality association in seven US cities Am J Epidemiol 2003 157 1074 1082 10.1093/aje/kwg096 12796043

[B2] Keatinge WR Donaldson GC Heat acclimatization and sunshine cause false indications of mortality due to ozone Environ Res 2006 100 387 393 10.1016/j.envres.2005.08.012 16221472

[B3] Medina-Ramon M Zanobetti A Schwartz J The effect of ozone and PM_10 _on hospital admissions for pneumonia and chronic obstructive pulmonary disease: A national multicity study Am J Epidemiol 2006 163 579 588 10.1093/aje/kwj078 16443803

[B4] Curriero FC Heiner KS Samet JM Zeger SL Strug L Patz JA Temperature and mortality in 11 cities of the eastern United States Am J Epidemiol 2002 155 80 87 10.1093/aje/155.1.80 11772788

[B5] Anderson BG Bell ML Weather-related mortality: How heat, cold and heat waves affect mortality in the United States Epidemiology 2009 20 205 213 10.1097/EDE.0b013e318190ee08 19194300 PMC3366558

[B6] Mireku N Wang Y Ager J Reddy R Baptist A Changes in weather and the effects on pediatric asthma exacerbations Ann Allergy Asthma Immunol 2009 103 220 224 10.1016/S1081-1206(10)60185-8 19788019

[B7] Marks GB Colquhoun JR Girgis ST Koski MH Treloar AB Hansen P Downs SH Car NG Thunderstorm outflows preceding epidemics of asthma during spring and summer Thorax 2001 56 468 471 10.1136/thorax.56.6.468 11359963 PMC1746065

[B8] U.S EPA Air Quality Criteria for Ozone and Related Photochemical Oxidants (Final) 2006 Washington, DC: U.S. Environmental Protection Agency EPA/600/R-05/004aF-cF

[B9] Stafoggia M Forastiere F Faustini A Biggeri A Bisanti L Cadum E Cernigliaro A Mallone S Pandolfi P Serinelli M Tessari R Vigotti MA Perucci CA EpiAir Group Susceptibility factors to ozone-related mortality: A population-based case-crossover analysis Am J Respir Crit Care Med 2010 182 376 384 Epub 2010 Mar 25 10.1164/rccm.200908-1269OC 20339147

[B10] Strickland MJ Darrow LA Klein M Flanders WD Sarnat JA Waller LA Sarnat SE Mulholland JA Tolbert PE Short-term associations between ambient air pollutants and paediatric asthma emergency department visits Am J Respir Crit Care Med 2010 182 307 316 Epub 2010 Apr 8 10.1164/rccm.200908-1201OC 20378732 PMC2921597

[B11] Jerrett M Burnett RT Pope CA Ito K Thurston G Krewski D Shi Y Calle E Thun M Long-term ozone exposure and mortality N Engl J Med 2009 360 1085 1095 10.1056/NEJMoa0803894 19279340 PMC4105969

[B12] Ruidavets JB Cournot M Cassadou S Giroux M Meybeck M Ferrières J Ozone air pollution is associated with acute myocardial infarction Circulation 2005 111 563 569 10.1161/01.CIR.0000154546.32135.6E 15699276

[B13] Smith RL Xu B Switzer P Reassessing the relationship between ozone and short-term mortality in U.S. urban communities Inhal Toxicol 2009 21 37 61 10.1080/08958370903161612 19731973

[B14] Middleton N Yiallouros P Kleanthous S Kololotroni O Schwartz J Dockery D Demokritou P Koutrakis P A 10-year time-series analysis of respiratory and cardiovascular morbidity in Nicosia, Cyprus: The effect of short-term changes in air pollution and dust storm Environ Health 2008 7 39 10.1186/1476-069X-7-39 18647382 PMC2517071

[B15] Kinney P Climate Change, Air Quality, and Human Health Am J Prev Med 2008 35 459 467 10.1016/j.amepre.2008.08.025 18929972

[B16] Balbus JM Malina C Identifying Vulnerable Subpopulations for Climate Change Health Effects in the United States J Occup Environ Med 2009 51 33 37 10.1097/JOM.0b013e318193e12e 19136871

[B17] O'Neill MS Ebi KL Temperature extremes and health: Impacts of climate variability and change in the United States J Occup Environ Med 2009 51 13 25 10.1097/JOM.0b013e318173e122 19136869

[B18] Bernard SM Samet MJ Grambsch A Ebi KL The potential impacts of climate variability and change in air pollution-related health effects in the United States Environ Health Perspect 2001 109 199 209 11359687 10.1289/ehp.109-1240667 PMC1240667

[B19] Civerolo KL Mao HT Rao ST The airshed for ozone and fine particulate pollution in the eastern United States Pure Appl Geophys 2003 160 81 105 10.1007/s00024-003-8767-6

[B20] Rao ST Ku JY Berman S Zhang K Mao H Summertime characteristics of the atmospheric boundary layer and relationships to ozone levels over the eastern United States Pure Appl Geophys 2003 160 21 55 10.1007/s00024-003-8764-9

[B21] Kalkstein LS Greene JS An evaluation of climate/mortality relationships in large US cities and the possible impacts of climate change Environ Health Perspect 1997 105 84 93 10.1289/ehp.9710584 9074886 PMC1469832

[B22] Sheridan SC The redevelopment of a weather-type classification scheme for North America Int J Climatol 2002 22 51 68 10.1002/joc.709

[B23] Pope CA Kalkstein LS Synoptic weather modeling and estimates of the exposure-response relationship between daily mortality and particulate air pollution Environ Health Perspect 1996 104 414 420 10.1289/ehp.96104414 8732952 PMC1469320

[B24] Smoyer KE Kalkstein LS Greene J Ye H The impacts of weather and pollution on human mortality in Birmingham, Alabama, and Philadelphia, Pennsylvania Int J Climatol 2000 20 881 897 10.1002/1097-0088(20000630)20:8<881::AID-JOC507>3.0.CO;2-V

[B25] Kysely J Huth R Heat-related mortality in the Czech Republic examined through synoptic and 'traditional' approaches Clim Res 2004 25 265 274

[B26] Sheridan SC Kalkstein AJ Kalkstein LS Trends in heat-related mortality in the United States, 1975-2004 Nat Hazards 2009 50 145 160 10.1007/s11069-008-9327-2

[B27] Davis RE Normile CP Sitka L Hondula DM Knight DB Gawtry SP Stenger PJ A comparison of trajectory and air mass approaches to examine ozone variability Atmos Environ 2010 44 64 74 10.1016/j.atmosenv.2009.09.038

[B28] McGregor GR Walters S Wordley J Daily hospital respiratory admissions and winter air mass types, Birmingham, UK Int J Biometeorol 1999 43 21 30 10.1007/s004840050112 10466017

[B29] Huth R Beck C Philipp C Demuzere A Ustrnul Z Cahynova M Kysely K Tveito O Classifications of atmospheric circulation patterns: Recent advances and applications Ann NY Acad Sci 2008 1146 105 152 10.1196/annals.1446.019 19076414

[B30] Technology Transfer Network (TTN) Air Quality System (AQS) http://www.epa.gov/ttn/airs/airsaqs/detaildata/downloadaqsdata.htm

[B31] Spatial Synoptic Classification http://sheridan.geog.kent.edu/ssc.html

[B32] Draxler RR Hess GD Description of the HYSPLIT_4 modeling system for trajectories, dispersion and deposition 1997 Silver Spring, MD: NOAA Tech Memo ERL ARL-224 NTIS PB98-116593

[B33] Hastie TJ JM Chambers and TJ Hastie Generalized additive models Statistical Models in S 1992 Wadsworth & Brooks/Cole Chapter 7 of

[B34] Zanobetti A Schwartz J Air pollution and emergency admissions in Boston, MA J Epidemiol Community Health 2006 60 890 895 10.1136/jech.2005.039834 16973538 PMC2566060

[B35] Forastiere F Stafoggia M Tasco C Picciotto S Agabiti N Cesaroni G Perucci CA Socioeconomic status, particulate air pollution, and daily mortality: Differential exposure or differential susceptibility Am J Ind Med 2007 50 208 216 10.1002/ajim.20368 16847936

[B36] Wong TW Wun YT Yu TS Tam W Wong CM Wong AHS Air pollution and general practice consultations for respiratory illnesses J Epidemiol Community Health 2002 56 949 950 10.1136/jech.56.12.949 12461117 PMC1756999

[B37] Sheridan SC North American weather-type frequency and teleconnection indices Int J Climatol 2003 23 21 45

[B38] Bloomer BJ Stehr J Piety C Salawitch R Dickerson R Observed relationships of ozone air pollution with temperature and emissions Geophys Res Lett 2009 36 L09803

